# Young, female and scientist: exploring barriers, challenges and opportunities

**DOI:** 10.1002/2211-5463.13972

**Published:** 2025-01-24

**Authors:** Nohelly Derosiers, Eline Bernaerts, Jessica L. Braun, Victoria Pozo Garcia, Radosveta Gencheva, Ana Paredes Garcia, Ioannis Tsagakis

**Affiliations:** ^1^ Department of Systems Pharmacology and Translational Therapeutics, Perelman School of Medicine University of Pennsylvania Philadelphia PA USA; ^2^ Department of Microbiology, Immunology and Transplantation Rega Institute for Medical Research, KU Leuven Belgium; ^3^ Department of Kinesiology Brock University St. Catharines Canada; ^4^ Division of Molecular and Computational Toxicology, Department of Chemistry and Pharmaceutical Sciences, Institute of Molecular and Life Sciences (AIMMS) Vrije Universiteit (VU) Amsterdam The Netherlands; ^5^ Department of Medical Biochemistry and Biophysics Karolinska Institutet Solna Sweden; ^6^ Wellcome Sanger Institute Hinxton UK; ^7^ FEBS Open Bio Editorial Office Cambridge UK

**Keywords:** academic culture, diversity, ECR, female scientists, gender bias, women in science

## Abstract

Different societal, systemic and personal barriers exist at various stages along a female researcher's career that can potentially undermine their success. The equation for women to reach higher positions in STEM is a multivariable one, and while there has been considerable progress towards addressing some of these compared with the past, current solutions are inadequate and do not address all facets. Here, we asked female winners of the *FEBS Open Bio* poster prize about their experiences regarding barriers they have faced at the predoctoral and postdoctoral stages, their opinions on how these can be addressed and their advice to new students entering a PhD degree.

## Graphical abstract

Societal, systemic and personal barriers exist at various stages along a female researcher's career that can potentially undermine their success. We asked female winners of the *FEBS Open Bio* poster prize about their experiences regarding barriers they have faced at the predoctoral and postdoctoral stages, their opinions on how these can be addressed and their advice to new PhD students.

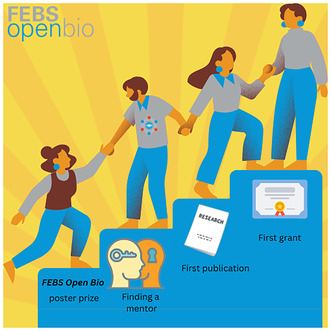

**Abbreviations**
DEI: diversity, equality and inclusion
ECR: early career researcher
STEM: science, technology, education and mathematics

## What are some of the barriers that you have faced in advancing your career as a female early career researcher (ECR)?

**ND:** As a black immigrant woman and early career researcher, I am reminded of the nature of our field any time I walk into a room. While it can be mentally challenging to know that I can be judged on factors other than my work, I choose to control what I can, staying motivated to break through any ceiling that might be placed on my success.

**EB:** One barrier that I have faced is the perception that men's opinions are more trustworthy and valuable than women's, which leads to situations where our opinions are less valued.

**JB:** Standing up to gender bias and inequalities, even in small ways, seems to go hand in hand with the thoughts ‘How will my speaking up negatively affect my career or change my colleagues' opinions of me?’ These thoughts have certainly stopped me in the past. In my current position, I am appreciative to be surrounded by many incredible women, but this can sometimes lead to a denial of the existence of barriers towards women in STEM. ‘Favouring’ of women with regards to hiring and grants doesn't eradicate the biases that persist. Instead, it has created the opinion that ‘you only got that position/grant because you're a woman’, leading us to believe that we don't deserve what we've worked so hard for, and that we must work twice as hard just to earn the respect of our colleagues. Another barrier revolves around conflict resolution. It can be difficult to raise valid concerns without the feeling of coming across as ‘naggy’, ‘catty’ or ‘too emotional’. Like a snowball effect, the process of conflict resolution then becomes skewed towards treating concerns as more of a personal issue than a workplace issue leading to a lack of resolution, feeling disrespected, discouraged and wanting to remain agreeable. A final note I'd like to bring attention to regarding barriers and advancing careers as a female ECR is the fact that being passionate about increasing diversity also means taking on substantial roles beyond research. For example, dedicating time to DEI‐related events means time taken away from research or from leisure.

**VPG:** During my PhD studies in the Netherlands, I perceived a reduction in gender inequality. However, both genders are not equally represented in higher academic positions (e.g. professorships). Addressing this gap is crucial for providing role models for junior researchers and inspiring future generations.

**RG:** I feel obliged to preface this by saying that I am lucky to have moved within EU countries and to have fair skin—these privileges are not to be underestimated.

I haven't been taken as seriously as my male colleagues, in all work‐related situations, from less formal group meetings to plenary discussions at conferences. I had my poster (poorly) explained to me at a conference and vehemently criticized without being let to speak at all to explain any misunderstandings. In another interaction, a male ECR complained to me how difficult it is to get funding nowadays because of the ‘silly’ quotas for female researchers and how unfortunate it is that he is being ‘forced’ to find women to collaborate with in his latest projects. I and several other doctoral students I know have had a hard time finding female examinators for the thesis committee because there are fewer women with the formal academic career qualifications required.

**APG:** To be honest, during my PhD and postdoc, I wouldn't say I have personally experienced inequality (against my male PhD and postdoc peers). However, I have noticed the imbalance in faculty positions, which also contributes to a lack of role models. It is important for female early career researchers to feel that there are equitable opportunities to attain leadership positions regardless of gender.

## How much support is there to overcome these challenges and how easily can it be accessed?

**ND:** It's no secret that available resources vary from institution to institution. Personally, being part of the Ernest E. Just Biomedical Society at UPenn has provided me with a sense of community. The many seminars geared towards professional development where speakers from underrepresented backgrounds talk about their journeys post‐PhD have been a continued source of inspiration.

**EB:** I have been very lucky to be mentored by a PI, who promotes and raises awareness around equity. Through her mentorship, I have gained confidence in my abilities and opinions, which has helped me assert myself in situations where my contribution might otherwise have been undervalued.

**JB:** With regard to locally organized support, such as at my institution, there is an annual Women in STEM event that always has great speakers and workshops that are free to attend. Beyond that though, most of the impactful support has come from resources that I have personally sourced, including organizations, such as Million Women Mentors, the Association for Women in Science and 500 Women Scientists. I have had several fantastic female mentors, both in and outside of STEM, that have been invaluable resources for me with regards to building confidence, asking questions or advice and being a source of support. I recognize that I'm lucky to have had such great female mentors available to me and that not everybody has that opportunity which is why I'm actively trying to create resources for others. A few years ago, I took on starting and running my local chapter for a Canadian organization called Girls SySTEM Mentorship. This programme, through pairing young female students with STEM professionals, aims to build a space for encouragement, give students the tools to understand and explore STEM, and provide the opportunity for these girls to make informed choices about their futures.

**VPG:** Personally, I found valuable support by connecting with fellow PhD students and researchers. I was able to receive and give fresh perspectives through discussions, brainstorming sessions and collaborative planning meetings, creating a sense of community and mutual encouragement.

**RG:** It has been a ‘pull yourself by your bootstraps’ kind of situation. I wouldn't say that there has been particular help or support beyond numerous commiseration talks with colleagues who have ended up in similar situations a little too often for comfort.

**APG:** I think it really depends on the context. Based on my experience in Spain, there are many female researcher networks you can join, but I always suggest finding a senior female researcher you trust who can mentor you.

For instance, I have been involved in several mentoring programmes specially for female ECRs (e.g. MECUSA). I think that the best asset you may have is to create your own network of female role models, across all career stages.

## How has the *FEBS Open Bio* poster prize award helped you/your career?

**ND:** Winning a *FEBS Open Bio* poster prize definitely made me feel more confident in my ability to communicate my work, both graphically and orally. It felt good to know that my poster stood out among the many amazing ones that were presented!

**EB:** Winning the *FEBS Open Bio* Poster prize has contributed to both my personal and professional growth. This prize has brought attention to me as a female ERC, which has been a meaningful boost to my self‐esteem. Knowing my work is valued has strengthened my belief in its importance. This recognition encouraged me to present my research work at more conferences, allowing to expand my professional network and gain valuable feedback from the research community.

**JB:** Winning a *FEBS Open Bio* poster prize was a big achievement for me early in my research career. I won this award at the 2022 European Muscle Conference which was one of my first international conferences and one of my first conferences post‐COVID‐19. For that reason, there was a lack of confidence going in, as well as some imposter syndrome, so being recognized with an award helped wash that away. Moving forward as a recipient of this award, I felt more confident in myself and my expertise which pushed me to apply to and compete for more awards at conferences and each experience has been a valuable lesson. Beyond the conference setting, this award helped me realize that I really do love research and getting to share my work with others, take feedback as it comes and understand the collaborative nature of good science. At the end of the day, we all have the same goal and sharing data, ideas and being open to criticism is what ensures we keep moving forward as a research community.

**VPG:** It certainly reinforced my confidence in my work and how it is perceived by others. I believe that discussions in science about your own research topic are essential to improve confidence, performance and critical thinking.

**RG:** The effect it had was honestly bigger than expected. More people noticed me at other conferences and paid more attention to what I had to say. Of course, it was a good confidence boost, and I was thrilled that people from adjacent fields thought my project's flavour of redox biology was interesting and promising.

**APG:** Winning the *FEBS Open Bio* poster prize boosted my self‐esteem when pitching the project, given my expertise was not in mitochondrial biology. Having this recognition from mitochondrial experts truly made my supervisor and I believe in the project and reassured us that we were heading in the right direction.

## What advice would you give to someone who is just starting out their PhD/scientific career, and what do you wish you knew when you were first starting out?

**ND:** Laboratory culture is of the utmost importance. Science research will inevitably present its highs and lows. When the lows come, being surrounded by mentors and peers who create an uplifting environment can greatly boost one's ability to persevere.

**EB:** Surround yourself with people who encourage and support your scientific career, and unlock your limitless potential.

**JB:** Ensure that you have something to keep you motivated. Whether it be your passion for what you're researching, having a specific career aspiration, or something else entirely, having motivation throughout your degree will help you through the days when you feel like you've made the wrong decision, or that nothing is working out. It's important to remember it's not always about ‘what’ you're doing, but the ‘why’. Second, setting realistic expectations or goals throughout the PhD will provide something to reach for, but won't leave you with continuous disappointment. I personally struggled early on with setting extremely high personal expectations which left me with either a poor work/life balance or leaving myself feeling like a failure if I didn't achieve my timeline. While it's very important to have goals and general timelines, it's equally as important to ensure they are achievable and leave room for flexibility—it is already a taxing field to enter, so there's no need to make it harder for yourself! Third, finding a strong support system through mentors, fellow grad students and family/friends will allow you to always have access to feedback, support, advice and encouragement. Finally, I can't stress enough the importance of setting boundaries. I have faced the consequences of over‐committing in the past, becoming overwhelmed and getting burnt out. Know your limits and don't be afraid to say no—another opportunity will come. Ensure to allocate time to rest and enjoy life, you'll be better for it!

**VPG:** The first piece of advice is to not give up. They probably started their career with a lot of motivation and passion for scientific knowledge, and they should have that very present. If things do not go as expected, it is okay. When in life do things go as planned? Well, in science, the same. The second piece of advice is to establish as many connections with fellow PhDs as possible, to have both professional and personal support during the scientific career. It will make the journey more joyful and easy. My third piece of advice is that at the end of the day it is your career, your research and your ideas. Pursue what you believe in and are passionate about. You are enough to make it work, and if you lack some knowledge to do so, you will learn!

**RG:** Choosing a supervisor and work environment which are a good fit for you personally is absolutely paramount, and I would argue is more important than committing to a field of research. In a sense, it can make or break your experience because a supportive environment will open so many more doors. Talk to people, try to be collaborative and open about ideas—you won't always be met with the same openness and enthusiasm on the other side, but I think you might be surprised by the opportunities which will present themselves to you if you keep at it.

**APG:** Having fun while pushing the boundaries of knowledge is such a privilege! To me, there is no point in doing science without that. Of course, there are rough days, but if you can keep feeling your passion even on those days, just keep going. Tomorrow will be another day. I wish I had known the power of collaboration and how crucial is to establish your peers' network!

## Conflict of interest

The authors declare no conflict of interest.

## Author contributions

All authors wrote and revised the manuscript.

